# Prediction of Speech Intelligibility by Means of EEG Responses to Sentences in Noise

**DOI:** 10.3389/fnins.2022.876421

**Published:** 2022-06-01

**Authors:** Jan Muncke, Ivine Kuruvila, Ulrich Hoppe

**Affiliations:** ^1^Department of Audiology, ENT-Clinic, University Hospital Erlangen, Erlangen, Germany; ^2^WS Audiology, Erlangen, Germany

**Keywords:** speech intelligibility, objective speech audiometry, EEG measures, speech tracking, auditory evoked potentials, speech in noise

## Abstract

**Objective:**

Understanding speech in noisy conditions is challenging even for people with mild hearing loss, and intelligibility for an individual person is usually evaluated by using several subjective test methods. In the last few years, a method has been developed to determine a temporal response function (TRF) between speech envelope and simultaneous electroencephalographic (EEG) measurements. By using this TRF it is possible to predict the EEG signal for any speech signal. Recent studies have suggested that the accuracy of this prediction varies with the level of noise added to the speech signal and can predict objectively the individual speech intelligibility. Here we assess the variations of the TRF itself when it is calculated for measurements with different signal-to-noise ratios and apply these variations to predict speech intelligibility.

**Methods:**

For 18 normal hearing subjects the individual threshold of 50% speech intelligibility was determined by using a speech in noise test. Additionally, subjects listened passively to speech material of the speech in noise test at different signal-to-noise ratios close to individual threshold of 50% speech intelligibility while an EEG was recorded. Afterwards the shape of TRFs for each signal-to-noise ratio and subject were compared with the derived intelligibility.

**Results:**

The strongest effect of variations in stimulus signal-to-noise ratio on the TRF shape occurred close to 100 ms after the stimulus presentation, and was located in the left central scalp region. The investigated variations in TRF morphology showed a strong correlation with speech intelligibility, and we were able to predict the individual threshold of 50% speech intelligibility with a mean deviation of less then 1.5 dB.

**Conclusion:**

The intelligibility of speech in noise can be predicted by analyzing the shape of the TRF derived from different stimulus signal-to-noise ratios. Because TRFs are interpretable, in a manner similar to auditory evoked potentials, this method offers new options for clinical diagnostics.

## 1. Introduction

According to the latest World Health Organization world report on hearing, more than 430 million people suffer from hearing impairment and hearing loss (HL) is the third largest cause of disability during a person's lifetime (World Health Organization, 2021). Even mild HL may lead to communication deficits caused by impaired speech intelligibility, particularly in noisy conditions (Dubno et al., 1984). The primary aims of audiological diagnosis are (1) to identify the underlying pathology for HL, and (2) to quantify the amount of HL and its effects on communication in daily life.

A number of objective audiological tests—such as measurements of acoustic impedance, of otoacoustic emissions, and of auditory evoked responses—are aimed at localizing the source of the deficiency. The determination of the degree of HL is usually carried out by subjective tests, in which the test person has to cooperate actively and to indicate the perception of auditory stimuli. The problem gets worse when we want to quantify the impact of a person's HL on their social life. Most often, this is carried out by using speech tests in quiet and in noise. These tests are usually performed by counting the number of words repeated correctly from certain test lists in a specific acoustic situation. Speech intelligibility measurements, especially in noise, require a high level of vigilance and cooperation. Hence, methods to measure the intelligibility of speech in noise (SiN) without active cooperation by the subjects are highly desirable in clinical audiological diagnosis.

A commonly used objective method for diagnosis is the measurement of auditory evoked potential (AEP) using electroencephalography (EEG). From repeated EEG measurements synchronized with the beginning of the auditory stimuli, as tone-bursts or clicks, specific waveforms can be derived. EEG changes caused by auditory processing can last up to 500 ms after the stimulus onset (Burkard et al., 2007). The AEPs are classified as short (1–10 ms, cochlear), middle (10–50 ms, auditory brainstem), and long (50–300 ms, auditory cortex) latency. Long latency AEP possesses a waveform structure characterized by the peaks P1 (positive, around 50 ms), N1 (negative, around 100 ms), P2 (positive, around 150 ms), and N2 (negative, around 300 ms). Compared with the background EEG, AEPs are considerably smaller, with a signal to noise ratio (SNR) of about -10 dB (Hoppe et al., 2001). Hence, to extract an AEP, the stimulus needs to be presented over several iterations (trials), and the EEG signals should be averaged over these trials (Burkard et al., 2007). In the past few decades it has been shown that short speech stimuli, presented as syllables, can be used for AEP measurements (Burger et al., 2009; Digeser et al., 2009). However, it was observed that the latency of the N1 peak and the P2 peak differs between syllables depending on the stimulus onset time (Sharma and Dorman, 1999), making it difficult to compare or average them. Moreover, presenting the speech stimulus repeatedly over several trials may not be the best choice because of altered speech processing caused by repetition. The analysis becomes more challenging when cortical responses to continuous speech are measured. Contrarily, to short and isolated stimuli, a continuous and time varying stimulus that elicits a lot of concatenated responses, can not be averaged over several trials. Hence, an alternative method to estimate cortical responses to continuous speech would be highly attractive.

Different methods that circumvent the aforementioned constraints in estimating the response to continuous speech by using ridge regression (Machens et al., 2004), boosting (David et al., 2007), or the Bayesian principle (Kuruvila et al., 2020) have been described recently. Roughly, these methods consider speech evoked EEG responses as a linear convolution of the speech envelope and an unknown impulse response plus internal noise. The impulse response function is determined by the auditory system and is referred to as the temporal response function (TRF), or the forward model (Speech → EEG). The method to estimate the TRF used in this paper is a regularized ridge regression based on the least-squares estimation principle (Lalor et al., 2006, 2009). Numerous studies have used the intensity of the stimulus as the acoustic cue to estimate the TRF (Aiken and Picton, 2008; Lalor and Foxe, 2010; Ding and Simon, 2012a; Mesgarani and Chang, 2012), and intensity is represented to a good approximation by the acoustic envelope of the stimulus. Since TRF describes the impulse response, once determined, it can be used to predict the EEG response to any input signal. Conversely, the response function could be estimated in the backward direction (EEG → Speech). The estimated backward model could then be used to reconstruct the stimulus from the EEG, accordingly this method is known as stimulus reconstruction (O'Sullivan et al., 2014). The accuracy of prediction, or reconstruction, can be estimated as the correlation coefficient between the derived and the predicted EEG, or between the original and the reconstructed envelope.

The relationship between recognition of speech in noise and the accuracy of stimulus reconstruction has been the focus of several recent studies (Etard and Reichenbach, 2019; Iotzov and Parra, 2019; Zou et al., 2019). Vanthornhout et al. (2018) investigated stimulus reconstruction accuracy for measurements with different SNRs using EEG measurements from 64 electrodes. They found a strong correlation between speech envelope reconstruction and actual speech envelopes. The method was refined by Lesenfants et al. (2019) using a forward model and the prediction accuracy of selected electrodes. They were able to predict individual's speech recognition thresholds (i.e. the SNR at which 50 % is understood, SRT_50_) with an accuracy of 1–2 decibels. In all of the studies mentioned, EEG measurements were used for speech in quiet to estimate the TRF, and these TRFs were applied to SiN measurements. Hence, those authors did not take account of possible changes in TRF morphology caused by the noise, even though such changes are well known (Zou et al., 2019; McHaney et al., 2021). Accou et al. (2021) chose a nonlinear approach by using a convolutional model, trained on speech in quiet, to solve a match—mismatch paradigm at different stimulus SNR and predict the SRT_50_. As the convolutional model can bee seen as a black-box, it is hardly physiologically interpretable.

The aim of this study was to determine the TRFs associated with speech signals in noise, at different SNRs covering the complete transition region of speech intelligibility. The shape of the individual TRFs was used to extract intelligibility relevant parameters as an objective and physiologically interpretable measure of speech intelligibility. By comparing these parameters with individual speech intelligibility scores, we aimed to derive EEG based measures of SiN. Finally, the combination of scalp electrodes and features yielding the highest correlation with changes of SNR were compared with subjective evaluation of speech intelligibility.

## 2. Materials and Methods

### 2.1. Participants

Eighteen normal hearing, right-handed German native speakers (6 male / 12 female) with a median age of 31 years (range 20–60 years) were recruited for this study. All subjects reported no history of neurological disorders and underwent an audiological examination including pure tone audiometry and otoscopy before the experiment. Normal hearing was defined as a maximum pure tone threshold of 25 dB HL for all octave frequencies between 0.5 and 4 kHz at the better ear. Additionally, the speech recognition for Freiburg monosyllabic words had to be 100% at a presentation level of 50 dB SPL.

### 2.2. Test Procedure

**Figure 1:**
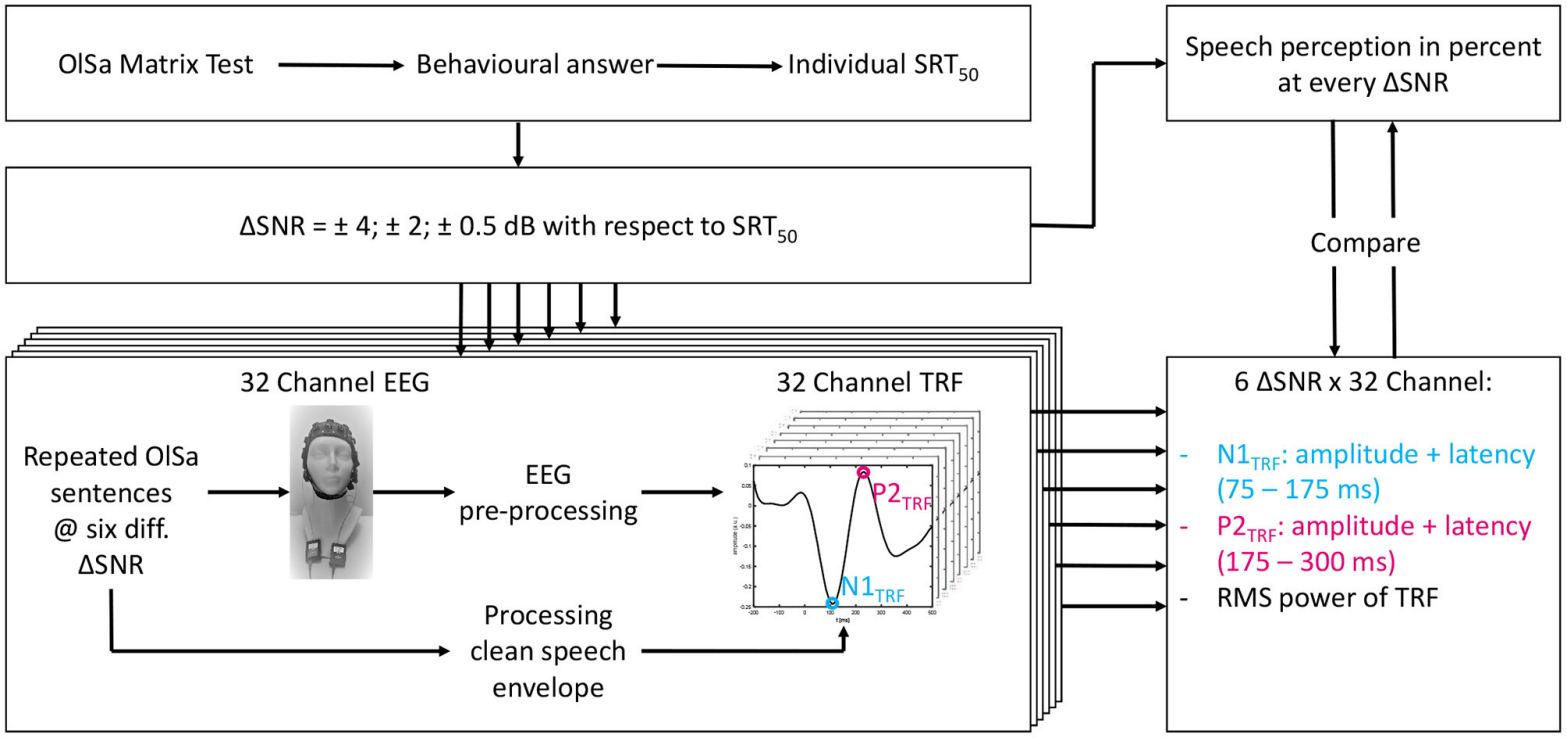
Scheme of the test procedure for each subject. First the individual SRT_50_ was obtained (upper left). The subsequent tests were all performed under six different ΔSNR conditions (defined below). TRF estimation is shown in the lower left and feature extraction in the lower right corner. Finally, features with highest correlation with speech intelligibility in per cent per ΔSNR condition were compared. All behavioral measurements took place on the first and all objective measurements on the second day.

Figure 1 displays an overview of the entire test procedure. Measurements were carried out on two different days in order to avoid effects of fatigue. On the first day, otological and audiological assessments were carried out. Thereafter, the behavioral SiN measurements were performed by using the Oldenburg Sentence test (Wagener et al., 1999c). At first, the individual's speech recognition threshold, SRT_50_ (i.e. the SNR at which 50 % of the speech material can be repeated correctly) was measured by using an adaptive routine (Kollmeier et al., 2015) with constant speech level at 60 dB SPL. Thereafter, the speech recognition rate at each of six different SNRs below and above the individual SRT_50_ (SRT_50_ +4 dB, +2 dB, +0.5 dB, –0.5 dB, –2 dB, and –4 dB) was determined by evaluating 20 sentences per condition. The masker noise was played without interruption during every test. According to the psychometric function of Wagener et al. (1999b), recognition rates of 93, 80, 60, 40, 20, and 6% are expected (Wagener et al., 1999c). The different SNR conditions will hereinafter be referred to as ΔSNR. With the aim of comparing theoretical and measured values, the psychometric function $f\left(SNR\right)=\frac{1}{1+exp\left(-\frac{SNR-SRT_{50}}{s}\right)}$ of Wagener et al. (1999b), with s indicating the slope, was fitted to the evaluated intelligibility over all subjects by using a non linear least squares approach.

All stimuli were presented diotically *via* Etymotec ER-2 insert phones connected to a Fireface UC sound card (RME Haimhausen Germany). The insert phones were calibrated to a speech level of L_*s*_ = 60 dB with a 2 cc coupler. EEGs were recorded by using a 32-channel gTec USBAMP device with active electrodes (g.tec, Schiedlberg, Austria) and all the measurements took place in a soundproof and acoustically damped room. Analyses and results presented in this paper were carried out with MATLAB R2019b (MathWorks, Nattick, MA; USA) in combination with the EEGLAB Toolbox (Brunner et al., 2013; Iversen and Makeig, 2019) and SPSS 24 (IBM, New York, NY, USA).

#### 2.2.1. EEG Experiments

EEG measurements were performed on a single day, 1 week later. Subjects listened passively to sentences of the Oldenburg Sentence test (OlSa). For each ΔSNR condition the same 10 sentences of the OlSa content where put in a complete randomized order, with every sentence appearing 15 times. When presenting the sentences, they were concatenated per condition while the masking noise (randomly overlapped sentences of the OlSa content; Wagener et al., 1999a) was played without interruption. The speech level was kept fixed at a level L_*S*_ = 60 dB SPL in all trials, to avoid effects of stimulus intensity on the derived TRFs (Verschueren et al., 2021). The noise level was adjusted per condition. The mean interval between sentences was 1.4 s (ranging from 0.9 to 1.8 s) and as a result, the duration of the experiment per ΔSNR condition was about 9 min. In order to keep the vigilance constant over the test duration (Vanthornhout et al., 2019) and to reduce eye movement (Kong et al., 2014), subjects were allowed to watch a silent movie. After measurement under each condition short breaks were taken. EEG and stimuli were synchronized to the recording system at the beginning and at the end of each sentence. Electrodes were mounted according to the international 10–20 system: the reference electrode was placed at the right earlobe and ground was placed at the F9 location. EEG was recorded at a sampling rate of 4,800 Hz.

### 2.3. Data Processing

The quality and reliability of the estimated TRF depends on the upper and lower cut-off frequencies of the bandpass filter applied to the raw EEG-recordings. While some studies reported that the highest correlation between speech intelligibility and analyzed EEG was obtained when a filter in the delta band (1–4 Hz) was used (Ding and Simon, 2014; Etard and Reichenbach, 2019; Iotzov and Parra, 2019), other studies reported the highest correlation when the theta band (4–8 Hz) was used (Lesenfants et al., 2019). However, we found the most highly significant results when we applied a filter including the delta and theta bands, resulting in a frequency band from 1 to 10 Hz. The chosen range includes the mean matrix test syllable rate of 3.8 Hz (Wagener et al., 1999a), which is close to the cut off frequency between delta and theta band.

The EEG-recording was down sampled to 120 Hz. Subsequently the signal was band-pass filtered non causal, zero phase between 1 and 10 Hz by applying a Hamming windowed-sinc FIR filter, order 397. Finally, an independent component analysis was applied to the EEG recordings in order to reduce the influence of eye blinks (Mennes et al., 2010). The envelope of the speech signal was obtained by taking the absolute value of the speech signal followed by power law compression by 0.6 according to Biesmans et al. (2017). Afterwards, the speech signal was down sampled to to 120 Hz to apply the same band-pass filter as had been used for filtering the EEG recordings. The standard functions of the EEGLAB Toolbox (Brunner et al., 2013; Iversen and Makeig, 2019) were used for the preprocessing procedure.

### 2.4. TRF Using Regularized Linear Regression

For a given input stimulus *s* and the observed response *r*(*t*) at an EEG electrode, the TRF of the system can be approximated by a linear regression model (Lalor et al., 2006, 2009). Mathematically, the predicted response $\hat{r}$ at time t can be expressed as a linear convolution between the input stimulus *s* and the TRF for the specified time lags τ, such that


(1) $$\begin{array}{r} \hat{r}\left(t\right)=\underset{\tau }{\sum }TRF\left(\tau \right)s\left(t-\tau \right). \end{array}$$

The cost function *J*(*t*), which is defined as the squared error between the measured response and the predicted response, can be written as


(2) $$\begin{array}{r} J\left(t\right)=\underset{t}{\sum }{\left[r\left(t\right)-\hat{r}\left(t\right)\right]}^{2}. \end{array}$$

An optimum estimate of the TRF can be obtained by minimizing the cost function *J*(*t*) (Kay, 1993). At minima, the gradient of *J*(*t*) vanishes and the estimated TRF can be expressed as


(3) $$\begin{array}{r} TRF={\left(S^{T}S\right)}^{-1}S^{T}r. \end{array}$$

The columns of the correlation matrix *S* are generated from the time-lagged versions of the stimulus envelope *s*. As a result, the TRF may overfit to fast fluctuations of the specific data set, particularly in noisy conditions (Crosse et al., 2016). Hence, regularization is employed by penalizing the *L*_2_ norm of the solution (Lampe and Voss, 2013) in order to smooth the TRF. The regularized solution of (3) is given by


(4) $$\begin{array}{r} TRF_{reg}={\left(S^{T}S+\lambda I\right)}^{-1}S^{T}r, \end{array}$$

where λ is the regularization parameter and *I* is the identity matrix.

In our analysis, a single TRF was calculated at every electrode, for each ΔSNR condition and subject. Time lags τ considered to generate the correlation matrix *S* were chosen from –200 ms to 500 ms in steps of 8,3 ms. Since the regularization parameter λ has an effect on the shape and the amplitude of TRF, it was kept constant (λ = 2^15^) through out the analysis. The λ parameter was optimized iterative in order to maximize Spearman correlation (see Section 2.6). The TRF estimations were performed using the mTRF Toolbox for MATLAB (Crosse et al., 2016).

### 2.5. TRF Feature Extraction

From the evaluated data, we calculated a total of 192 TRFs for each subject, corresponding to 32 channels and six SNR conditions. Because a TRF can be interpreted similarly to slow AEP (Picton, 2013; Di Liberto et al., 2015; Fiedler et al., 2019; Kuruvila et al., 2021), peaks corresponding to the waves N1 and P2 of AEP can be detected. Here they are designated as N1_TRF_ and P2_TRF_ (see Figure 1). Five features (the absolute amplitude and latency of N1_TRF_, the amplitude and latency of P2_TRF_, and a windowed root mean square (RMS) value (Zou et al., 2019) were extracted for each TRF in order to analyze speech intelligibility. N1_TRF_ was determined as the center of the first local minimum in the latency range between 75 and 175 ms, identified by evaluating the first derivative. Similarly P2_TRF_ was identified as the first local maximum in a range from 175 ms to 300 ms. If no N1_TRF_ and P2_TRF_ were detected, latency was set to its maximum value and amplitude was set to zero. The RMS value was calculated as the root mean square of the TRF per EEG channel, after applying a time window to the TRF. The lower (0 to 100 ms) and the upper (108 to 200 ms) limits of the windows were optimized around the prominent N1 peak (Billings et al., 2013; Bidelman and Howell, 2016) for each electrode in order to achieve maximum Spearman correlation with ΔSNR over all subjects (see Section 2.6).

### 2.6. Statistical Analysis

We expected a lower amplitude and a higher latency of waves N1_TRF_ and P2_TRF_ with decreasing SNR (Mirkovic et al., 2019; Zou et al., 2019). Consequently, we expected smaller RMS values for lower SNRs. To find out which electrodes best fulfilled this expectation (e.g. higher SNR $\hat{=}$ lower latency; higher SNR $\hat{=}$ higher amplitude), the monotonicity was evaluated by calculating Spearman's ρ of ΔSNR and the investigated feature for each subject and electrode. Furthermore, for each electrode the mean over all subjects was determined. In order to test the hypothesis that ΔSNR conditions have the same mean, a Kruskal-Wallis test was performed for each electrode and feature. If the hypothesis was rejected, a Dunn-Bonferroni *post-hoc* test was performed for a pairwise comparison of ΔSNR conditions.

## 3. Results

### 3.1. Behavioral Test

**Figure 2:**
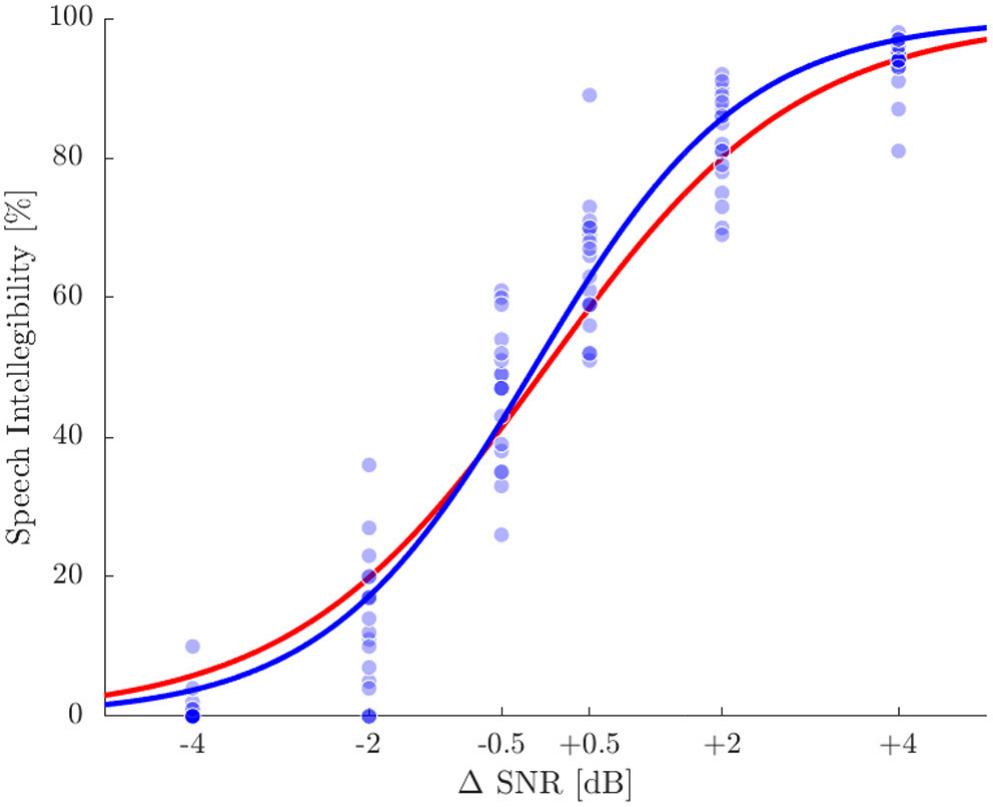
Speech intelligibility in percentage for the six ΔSNR conditions. Blue dots represent single measurements and the blue line displays a sigmoid function fitted to the single values. The red line represents the reference function according to Wagener et al. (1999c).

The individual SRT_50_ for the SiN test was used as the baseline for all tests for each subject. Mean SRT_50_ was –7.0 dB±0.9 dB (standard deviation), ranging from –4.9 to –8.9 dB. Individual speech intelligibility scores evaluated at the six ΔSNR conditions are shown in Figure 2 together with the sigmoid fit (Wagener et al., 1999b) and the reference psychometric function according to Wagener et al. (1999c). The root mean square error of actual values and the reference function was 6.9%, the largest difference was 29%.

### 3.2. TRF Analysis

**Figure 3:**
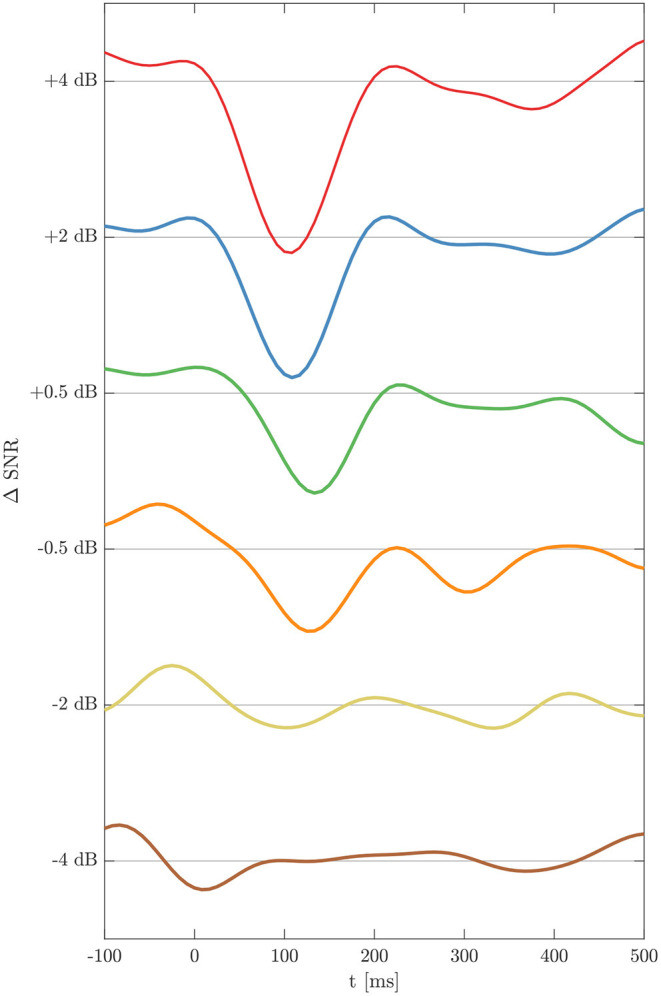
Temporal response functions for the six ΔSNR conditions, calculated as grand average over all subjects for electrode C3. TRFs exhibit peaks N1_TRF_ and P2_TRF_ down to a ΔSNR of –2 dB.

EEG measurements for six SNR × 32 electrodes × 18 subjects were performed resulting in estimations of 3456 TRFs which were further analyzed. Figure 3 shows the grand average for the TRFs from electrode C3 at ΔSNR from –4 to +4 dB. At the highest SNR a clear negative deflection at about 100 ms is seen followed by a positive deflection at about 200 ms. TRF morphologies and latencies are congruent with those obtained from tone evoked AEP measurements. With decreasing SNR, N1_TRF_ and P2_TRF_ amplitudes decrease while corresponding latencies increase.

#### 3.2.1. Amplitude and Latency for N1_TRF_

**Figure 4:**
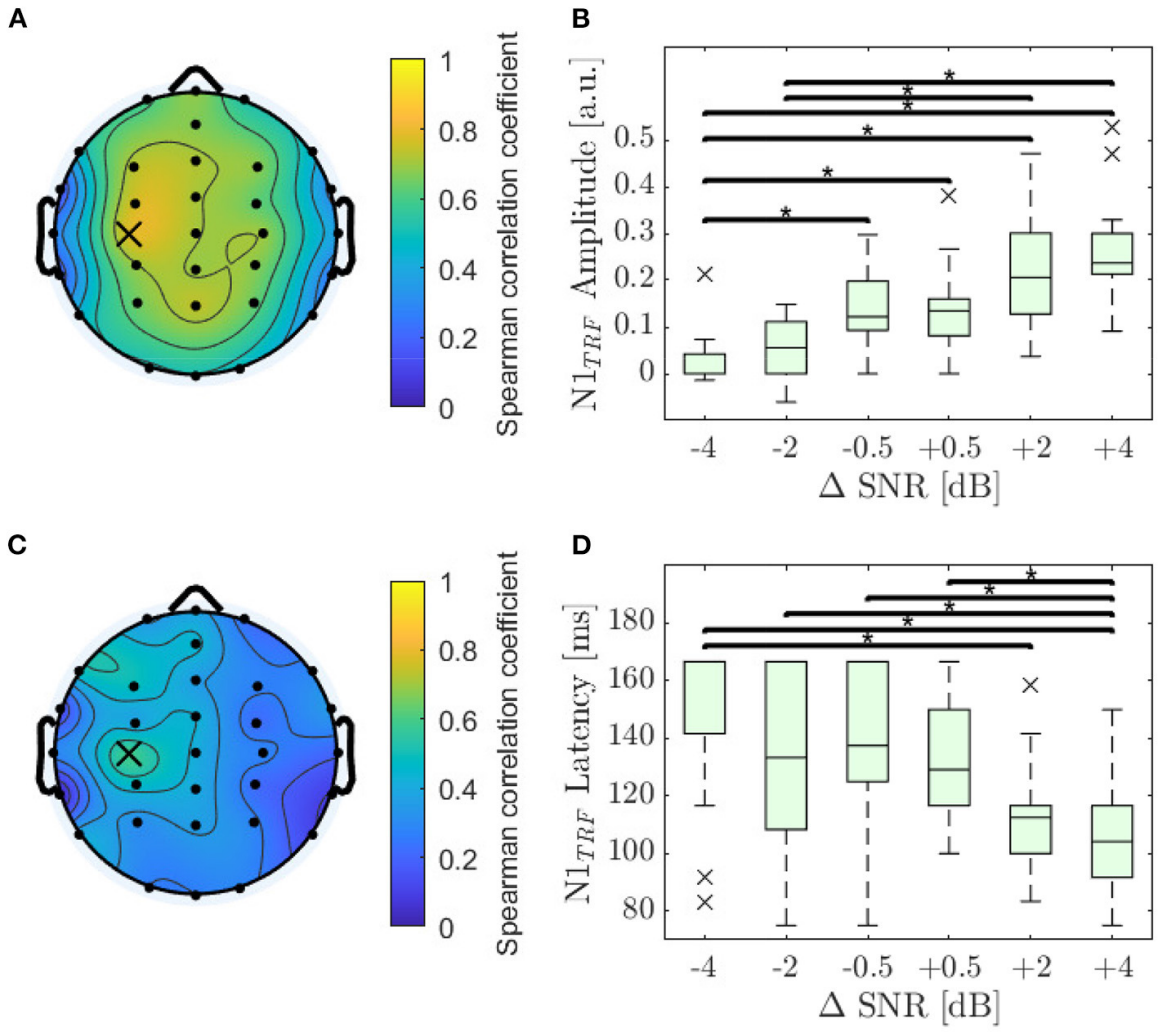
Analysis of the N1_TRF_. **(A)** Distribution of the monotonicity for amplitude of the N1_TRF_ evaluated by using Spearman's ρ, reaching its maximum value at electrode C3 (ρ = 0.78; *p* < 0.001). **(B)** Amplitude values for N1_TRF_ as a function of ΔSNR at electrode C3. Asterisks denote significant differences evaluated by a Dunn-Bunferroni *post-hoc* test. **(C)** Distribution of the absolute monotonicity for the N1_TRF_ latencies evaluated by using Spearman's ρ, reaching its maximum value at electrode C3 (marked with a cross; ρ = 0.48; *p* < 0.001). **(D)** Latencies for N1_TRF_ as a function of ΔSNR at electrode C3.

For each subject and electrode the Spearman correlation as described in Section 2.6 was calculated. The highest correlation for feature N1_TRF_ amplitude was found at electrode C3 (ρ = 0.78; *p* < 0.001). The distribution of Spearman's ρ is displayed in detail in Figure 4A and shows a slight dominance in the left hemisphere. Amplitude decreased significantly with decreasing SNR for 27 out of 32 electrodes, reaching highest significance on electrode C3 (χ^2^ = 53.3; *p* < 0.001). The values of N1_TRF_ amplitude obtained for the electrode with the highest correlation are shown in Figure 4B.

The highest correlation for N1_TRF_ latency was also found on electrode C3 (ρ = –0.59; *p* < 0.001). The distribution of Spearman's ρ for N1_TRF_ latency, displayed in detail in Figure 4C, shows a slight dominance of the left hemisphere. Latency increased significantly with decreasing SNR for 19 out of 32 electrodes, reaching the highest significance at electrode C3 (χ^2^ = 32.7; *p* < 0.001). The values of N1_TRF_ latency obtained for the electrode with the highest correlation are shown in Figure 4D.

#### 3.2.2. Amplitude and Latency for P2_TRF_

Spearman correlation for P2_TRF_ features reached a medium effect size, with a maximum for amplitude of ρ = 0.35 (*p* < 0.001) and a maximum for latency of ρ = –0.45 (*p* < 0.001). For the amplitude feature, the mean of amplitude was found to be the same across different conditions at all electrodes. Findings were the same for the latency feature except for three fronto-temporal electrodes (maximum χ^2^ = 20.1; *p* = 0.001). Therefore, detection of P2 seems not to be appropriate for evaluating speech intelligibility.

#### 3.2.3. Windowed RMS-Power

**Figure 5:**
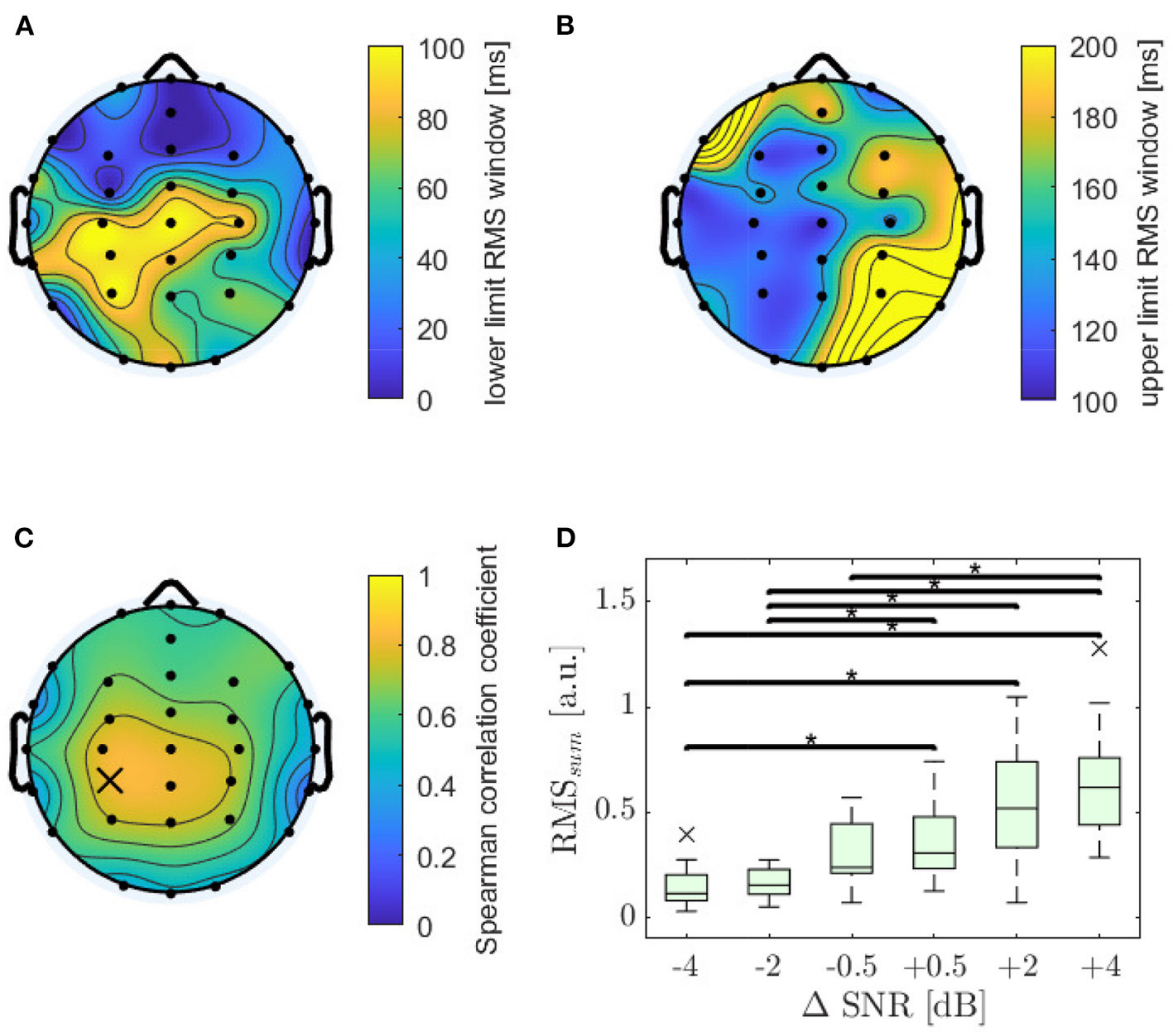
Analysis of wave RMS-power. **(A)** Optimal lower limits of the time window. **(B)** Optimal upper limits of the time window. **(C)** Distribution of monotonicity for windowed RMS-power, with an optimized window for each channel, evaluated using Spearman's ρ, which reached its maximum value at electrode CP3 (marked with a cross; ρ = 0.81; *p* < 0.001). **(D)** RMS_sum_ values as sum of RMS values for electrode C3, CP3, and CPz for six ΔSNR and 18 subjects. Asterisks show significant differences evaluated by a Dunn-Bonferroni *post-hoc* test, all with a strong effect.

The RMS value of every TRF was calculated by applying a rectangular time window to each TRF that was optimized in order to reach maximum correlation for each electrode. The resulting lower and upper limits of the windows are displayed in Figures 5A,B. The highest Spearman correlation for feature RMS was found at electrode CP3 (ρ = 0.81; *p* < 0.001). The distribution of Spearman's ρ is displayed in Figure 5C, showing a slight dominance of the left hemisphere. The RMS value decreased significantly with decreasing SNR for 30 out of 32 electrodes, reaching highest significance at electrode CPz (χ^2^ = 60.7; *p* < 0.001) for a window from 83 to 133 ms, followed by electrodes CP3 (92 ms to 125 ms), and C3 (83–125 ms). It can be observed that the highest Spearman's ρ are related to narrow windows around the N1 peak.

For further investigations the RMS values for the three electrodes CPz, CP3, and C3 were summed up to give RMS_sum_. RMS_sum_ achieved a strong Spearman correlation (ρ = 0.82; *p* < 0.001) and increased significantly with increasing SNR (χ^2^ = 60.0; *p* < 0.001). Detailed results of RMS_sum_ displayed in Figure 5D show increasing variance with increasing SNR.

#### 3.2.4. Features vs. Speech Intelligibility

A high Spearman correlation and a good ability to discriminate between the six ΔSNR conditions were achieved by the RMS_sum_ feature. The optimum time windows of the three electrodes for evaluating RMS_sum_ cover a narrow region close around the prominent N1_TRF_ peak. Finally, the simple RMS_sum_ feature was chosen as the single feature to be compared with speech intelligibility.

**Figure 6:**
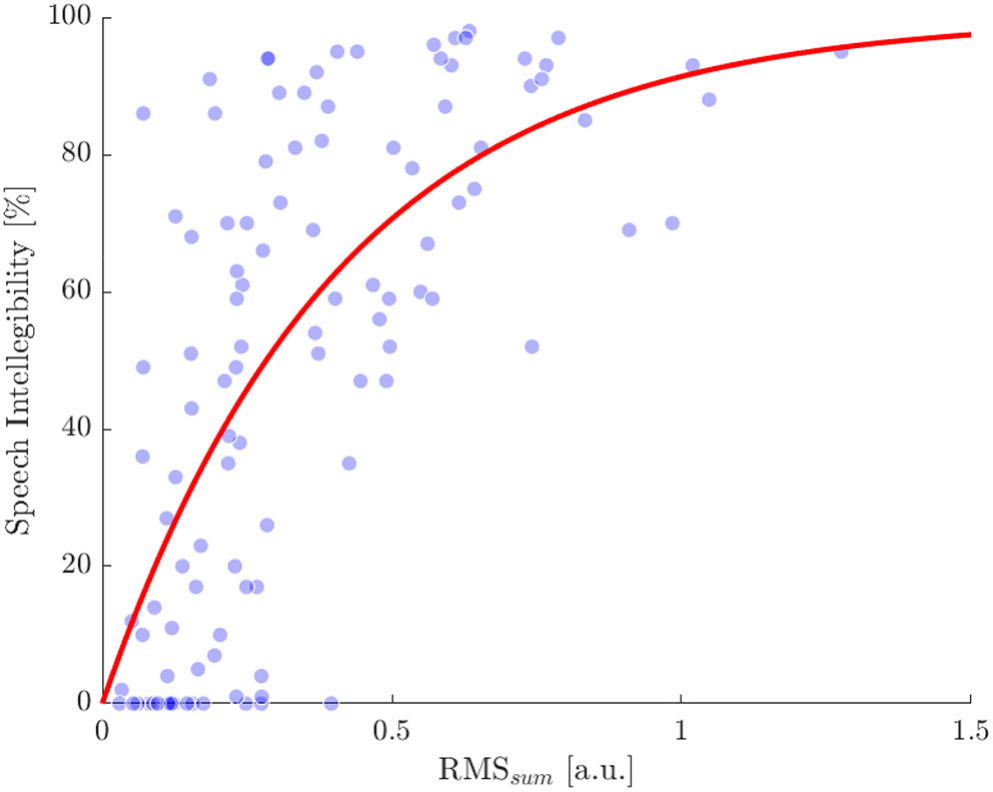
Comparison of speech intelligibility and the RMS_sum_ obtained for each ΔSNR condition and subject. Single values are indicated by blue dots. The red line shows a exponential function fitted to the single values. Root mean square error of the dots compared with the fitted curve is 25% and the RMS_sum_ value related to an intelligibility of 50% is 0.287.

To estimate individual SRT_50_ a psychometric function was fitted to the speech intelligibility evaluated at the different ΔSNRs. In the same way, to obtain the threshold related to an intelligibility of 50% (RMS_50_), we fitted the exponential function $Intelligibility\;=\;100\;\cdot \;\left(1\;-e^{\left(-b\cdot RMS_{sum}\right)}\right)\%$, with b being the optimized parameter, by using a non-linear least squares approach. The resulting function starts from zero % for a RMS_sum_ of zero and converges to 100% for higher values. When using the RMS_sum_ data of all subjects to fit the function, the RMS_50_ thus determined was 0.287 (Figure 6) and b was 2.415 with a 95% confidence interval from 2.03 to 2.799.

In order to prevent from over fitting, a leave one out method was applied to predict the individual SRT_50_ per subject. This was done by calculating the RMS_50_, without considering the RMS_sum_ data of the investigated subject. Subsequently, the value of ΔSNR at which RMS_sum_ exceeds the threshold RMS_50_ for the first time with increasing SNR and increasing RMS_sum_, indicates the predicted SRT_50_.

**Figure 7:**
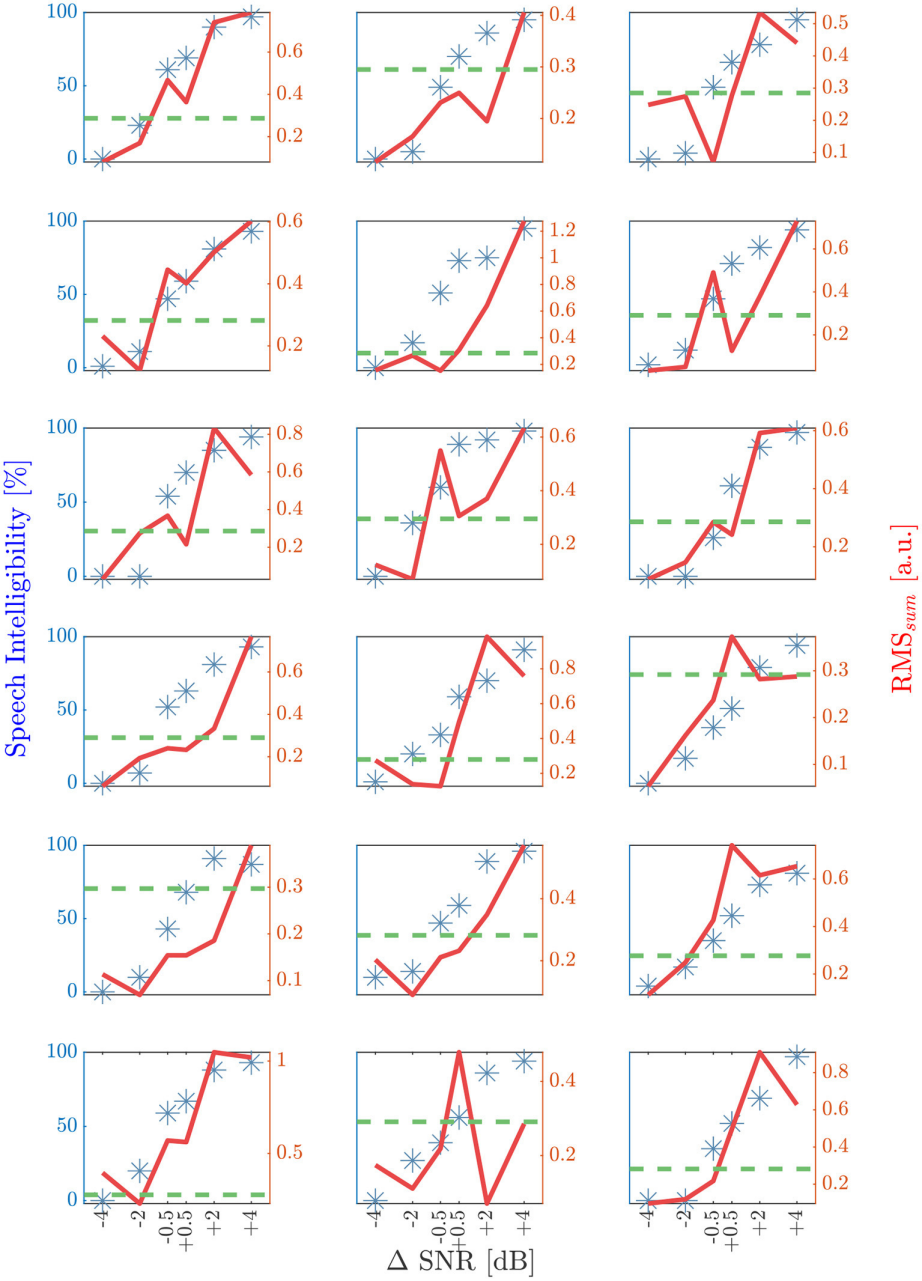
Results of the speech intelligibility and RMS_sum_ (ρ = 0.71; *p* > 0.001) for the six different ΔSNR conditions for each subject. A ΔSNR of zero dB corresponds to the behavioral SRT_50_. Blue stars are indicating the speech intelligibility in percentage and the red solid line shows the corresponding RMS_sum_ value. Green dashed horizontal line is representing the RMS_50_, determined by using a leave one out method per subject. The first intersection point with increasing RMS_sum_ and increasing SNR is located in a range of ±1 dB for seven and ±2 dB with respect to behavioral SRT_50_ for 16 out of 18 subjects.

Individual speech intelligibility and RM*S*_*sum*_ are compared with respect to ΔSNR in Figure 7, showing a strong correlation (Spearman's ρ = 0.71; *p* < 0.001). The mean deviation between predicted and behavioral SRT_50_ was 1.2 dB in a range from –1.8 dB to +3.1 dB.

## 4. Discussion

Reduced understanding of speech in noise is one of the first symptoms of hearing loss. All available tests to evaluate the intelligibility for an individual person are subjective tests that require subjects to repeat the words that they understood. First attempts to evaluate the SRT_50_ of a SiN test using EEG techniques was recently undertaken by Vanthornhout et al. (2018) and Lesenfants et al. (2019). In one procedure the reconstruction accuracy, and in the other procedure the prediction accuracy was chosen as predictor for SRT_50_. Regrettably, the authors of both studies used prediction or reconstruction accuracy to indicate intelligibility, without taking a closer look at changes in TRF morphology. Our results presented here show that changes in the TRF obtained at different SNRs can be used as a feature to predict individual SRT_50_ values for an SiN test. Because a TRF shows a waveform similar to an AEP (Ding and Simon, 2012b; Picton, 2013; Di Liberto et al., 2015; Fiedler et al., 2019), TRF morphology can be regarded as more amenable to physiological interpretation for clinical diagnostics than reconstruction or prediction accuracy. In order to find the features of TRF that are most strongly correlated with speech intelligibility we analyzed five features of TRF (N1_TRF_ amplitude, N1_TRF_ latency, P2_TRF_ amplitude, P2_TRF_ latency and RMS_sum_) evaluated for 18 subjects at six SNR values close to the respective individual SRT_50_.

The largest Spearman correlation with changes in SNR was found for the RMS_sum_ feature, using optimum windows, i.e., in the range from 82 to 133 ms. This range covers the latency of N1_TRF_, and N1_TRF_ amplitude also revealed a high Spearman correlation. In contrast, P2_TRF_ features showed only weak correlations with changes in SNR. Our findings correspond to those described in Billings et al. (2013) and Bidelman and Howell (2016), the authors of which investigated AEP evoked by syllables at different SNRs. The authors of both publications found the best correlation with speech intelligibility for the amplitude of wave N1, and the RMS_sum_ feature was finally used for comparison with speech intelligibility in our study.

We found the highest amplitudes for N1_TRF_ and RMS_sum_ in the central and fronto central scalp region, corresponding to the N1 wave of an AEP measurement that is known to be evoked in the planum temporale and Heschl's gyrus (Woods, 1995; Picton et al., 1999). Highest reconstruction accuracy is also often found in this scalp region (Etard and Reichenbach, 2019; Zou et al., 2019). Electrodes in the central and the fronto-central region have also been used by Lesenfants et al. (2019) to predict speech intelligibility. In contrast to those authors findings, the electrodes found in our study to be best suited for prediction of speech intelligibility were located in the central and the left-central scalp area (CPz, C3, and CP3). The distribution of monotonicity shows that the region crucial for speech intelligibility is concentrated slightly more in a posterior orientation with dominance of the left hemisphere. Our results correspond to the findings of other authors (Leff et al., 2008; Abrams et al., 2013) that the posterior superior temporal sulcus has a strong influence on processing speech intelligibility.

Even in a passive listening task the focal attention of the subject could vary with the perceived stimulus quality (e.g. SNR). The focal attention could affect the quality and amplitude of the derived TRF and probably has an effect on this study (Lesenfants and Francart, 2020). Speech in noise measurements for native and non-native speakers has been investigated by Etard and Reichenbach (2019) and by Zou et al. (2019). Those authors described similar behavior for both groups tested for reconstruction accuracy and power of a TRF by using the EEG theta band. Additionally, differences have been described between the groups only in the low-frequency EEG delta band (Etard and Reichenbach, 2019). We used a combination of both frequency bands and thus presumably included both effects. The correspondence of the location of best electrodes with earlier findings (Leff et al., 2008; Abrams et al., 2013) indicates a connection with speech intelligibility, whereas the presentation of the silent movie may have reduced slightly the subjects active listening and comprehension (Vanthornhout et al., 2019). On the contrary to repeating the same stimulus (Billings et al., 2013), we presented 10 different sentences in a random order and there was no a priori knowledge about the next sentence. Hence, listening to every sentence 15 times should have no effect due to the complete random order.

The theory of a network of speech comprehension (Leff et al., 2008; Abrams et al., 2013) and phonetic detection (Zatorre et al., 1992) located in different cortical areas and initialized at the posterior and anterior superior temporal sulcus corresponds to the electrodes used for RMS_sum_. It seems possible that our findings relate primarily to investigation of the auditory pathway to a certain stage depending on electrode localization. Not all cognitive requirements for a complete understanding of speech can be investigated by these measurements (Decruy et al., 2020a; Devaraju et al., 2021). Additionally, the chosen stimuli aim to imitate a matrix sentence test, resulting in periods between the single sentences, which only contain noise. Consequently, the sentence onset responses may have a slightly larger effect on the N1_TRF_ amplitude than the continuous speech (Brodbeck et al., 2018), suppressed with a higher noise level. Further investigations will be required to elucidate which stage of auditory processing is being examined here. This will be a prerequisite for using this method for differential diagnostics.

We achieved an RMS_sum_ level that was comparable across all subjects with increasing standard deviation for increasing SNR. The constancy of the TRF amplitude was supported by using active electrodes, which are less prone to quality of connection (Laszlo et al., 2014) and by applying a least squares approach with a constant regularization (Lalor et al., 2009; Power et al., 2012). Due to these facts the threshold for RMS_sum_ value in relation to speech intelligibility (RMS_50_) was evaluated. The intersection point of RMS_50_ and RMS_sum_ for individual subjects is in a range of ± 1 dB for 7 and ± 2 dB for 16 out of 18 subjects. Our results compare well with the prediction made in Lesenfants et al. (2019), even though the earlier study was based on a relatively technical approach while our results are more physiologically based. It seems possible to examine the physiological requirements for speech intelligibility in noise within a range of ± 2 dB using features extracted from TRF at different SNR values. Part of the variance can be explained by behavioral test-retest deviation (Wagener and Brand, 2005) and by the spread of behavioral speech intelligibility at fixed ΔSNR.

Many subjects reported that they got tired during the experiment. It would approve the practicability of the procedure if the time required could be reduced, maybe by increasing the steps between SNR conditions. Additionally, a reduction in the time required could reduce the influence of focal attention on the derived TRFs (Lesenfants and Francart, 2020). The procedure and the RMS_50_ value will have to be verified with younger and with hearing-impaired subjects, because the reconstruction accuracy is known to increase with hearing impairment (Decruy et al., 2020b), and AEPs are known to mature with age for children (Kummer et al., 2007).

## 5. Conclusion

We have shown that single features of the shape of TRFs are highly correlated with intelligibility for speech in noise. The detection of a RMS_sum_ level in the area of N1_TRF_ allowed at least approximate prediction of individual SRT_50_ values. Because TRFs are interpretable in a manner similar to AEPs, the method developed may offer new options for clinical diagnostics of difficulties during speech understanding in noise. With the aim of closer localizing the source of the deficit, it can be combined with other audiometric diagnostics starting from pure tone audiometry up to short and middle latency AEP which are related to the cochlear and the auditory brainstem. Additionally it can be possible to test subjects who may be less cooperative. It will be necessary to investigate to which stage of auditory processing the requirements for speech comprehension are examined and the method should be improved by fine tuning to make shorter trials possible. Additionally, further research should focus on testing the procedure with hearing-impaired and with younger subjects.

## Data Availability Statement

The raw data supporting the conclusions of this article will be made available by the authors, without undue reservation.

## Ethics Statement

The studies involving human participants were reviewed and approved by Ethics Committee at the University of Erlangen-Nuremberg (Ref. No. 449 18B). The patients/participants provided their written informed consent to participate in this study.

## Author Contributions

JM: conceived and designed the research, carried out the experiment, and performed the analysis. IK: verified the analytical methods. UH: conceived and designed the research and supervised the project. All authors discussed the results, contributed to the final article, and approved the submitted version.

## Funding

This work was funded by the Deutsche Forschungsgemeinschaft (DFG, German Research Foundation)—419293003—HO 2177/6.

## Conflict of Interest

IK was employed by the company WS Audiology. The remaining authors declare that the research was conducted in the absence of any commercial or financial relationships that could be construed as a potential conflict of interest.

## Publisher's Note

All claims expressed in this article are solely those of the authors and do not necessarily represent those of their affiliated organizations, or those of the publisher, the editors and the reviewers. Any product that may be evaluated in this article, or claim that may be made by its manufacturer, is not guaranteed or endorsed by the publisher.
